# The GIY-YIG Type Endonuclease Ankyrin Repeat and LEM Domain-Containing Protein 1 (ANKLE1) Is Dispensable for Mouse Hematopoiesis

**DOI:** 10.1371/journal.pone.0152278

**Published:** 2016-03-24

**Authors:** Juliane Braun, Arabella Meixner, Andreas Brachner, Roland Foisner

**Affiliations:** 1 Max F. Perutz Laboratories (MFPL), Department of Medical Biochemistry, Medical University of Vienna, Vienna Biocenter (VBC), Vienna, Austria; 2 IMBA-Institute of Molecular Biotechnology of the Austrian Academy of Sciences, Vienna, Austria; The University of Hong Kong, HONG KONG

## Abstract

Ankyrin repeat and LEM-domain containing protein 1 (ANKLE1) is a GIY-YIG endonuclease with unknown functions, mainly expressed in mouse hematopoietic tissues. To test its potential role in hematopoiesis we generated *Ankle1*-deficient mice. *Ankle1*^Δ/Δ^ mice are viable without any detectable phenotype in hematopoiesis. Neither hematopoietic progenitor cells, myeloid and lymphoid progenitors, nor B and T cell development in bone marrow, spleen and thymus, are affected in *Ankle1*^Δ/Δ^*-*mice. Similarly embryonic stress erythropoiesis in liver and adult erythropoiesis in bone marrow and spleen appear normal. To test whether ANKLE1, like the only other known GIY-YIG endonuclease in mammals, SLX1, may contribute to Holliday junction resolution during DNA repair, *Ankle1*-deficient cells were exposed to various DNA-damage inducing agents. However, lack of *Ankle1* did not affect cell viability and, unlike depletion of *Slx1*, *Ankle1*-deficiency did not increase sister chromatid exchange in Bloom helicase-depleted cells. Altogether, we show that lack of *Ankle1* does neither affect mouse hematopoiesis nor DNA damage repair in mouse embryonic fibroblasts, indicating a redundant or non-essential function of ANKLE1 in mouse.

## Introduction

Ankyrin-and LEM-domain containing protein 1 (ANKLE1) is a GIY-YIG-type endonuclease conserved from *Caenorhabditis elegans* [1] to man [2]. It is a member of the LAP2-Emerin-MAN1 (LEM)-domain containing protein family [3], defined by the presence of the LEM motif, a bi-helical structure that mediates interaction with the chromatin protein Barrier-to-Autointegration Factor (BAF) [4]. The mammalian genome contains seven genes encoding LEM-domain proteins, most of which are transmembrane proteins of the inner nuclear membrane. The well characterized integral membrane proteins of the LEM protein family, LAP2, Emerin, MAN1 and LEM2, are involved in a variety of nuclear functions, including nuclear architecture and mechanical stability, (hetero-)chromatin organization and the regulation of several signaling pathways and gene expression [3, 5–7].

In contrast to these inner nuclear membrane LEM proteins, ANKLE1 is soluble and contains a GIY-YIG endonuclease domain at its C-terminus that nicks and cleaves double stranded DNA in a BAF-dependent manner [2]. GIY-YIG motifs are found throughout evolution in all branches of life including phages, bacteria and eukaryotes, within selfish, mobile DNA elements, known as homing endonucleases that spread in the genome and were therefore integrated into various protein domains [8]. Nevertheless the only other so far identified GIY-YIG domain protein in mammals is the Holliday junction-resolving nuclease SLX1 [8] that is part of the homologous recombination (HR) repair pathway [9]. HR-mediated repair of DNA double strand breaks in somatic cells occurs during S/G2 phase, as it is dependent on the availability of sister chromatids serving as template for the damaged strand, where strand exchange leads to the formation of Holliday junctions [10]. While Holliday junctions between homologous chromosomes in meiotic cells can lead to cross-overs required for genomic diversity, somatic cells depend on error-free DNA double strand break repair through the dissolution of Holliday junctions by the BTR complex, consisting of Bloom helicase, topoisomerase IIIα, RMI1 and RMI2 [11]. SLX1 in association with the scaffolding protein SLX4 and a second endonuclease complex, MUS81/EME1, and the resolvase GEN1 resolve Holliday junctions that escape dissolution by the BTR complex, ensuring faithful chromosome segregation, however on the expense of introducing DNA breaks that can lead to both non-crossover and cross-over reactions [11].

Furthermore, it has been suggested that other still unknown components may be involved in the repair mechanisms, since *C*. *elegans* mutants lacking the known Holliday junction-resolving nucleases SLX1, MUS81 and GEN1, still retain resolvase activity [12]. Lem-3, the *C*. *elegans* orthologue of ANKLE1 may be a candidate [12], as worms expressing an endonuclease-dead version of Lem3 are more sensitive towards DNA damage caused by the DNA crosslinker cisplatin or UV-light [1]. The high redundancy of resolvases in the processing of Holliday junctions may also account for the observation that mice lacking SLX1 [13] or MUS81 [14] are viable, fertile and develop without gross defects. Nonetheless, on the cellular level, both *Slx1*^*-/-*^ and *Mus81*^*-/-*^ fibroblasts show increased sensitivity towards DNA interstrand crosslinking reagents.

In this study we generated an *Ankle1*-deficient mouse model to investigate the physiological role of ANKLE1 in mouse and its potential function in DNA repair. Based on our previous findings that *Ankle1* is predominantly expressed in hematopoietic tissues in human, we hypothesized that ANKLE1 may primarily function in hematopoiesis and we performed a comprehensive phenotypic analyses of the hematopoietic system in *Ankle1*^Δ/Δ^*-*mice.

## Results

**Fig 1:**
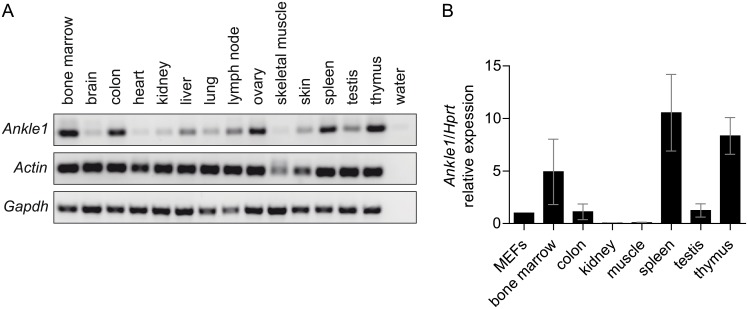
Expression of *Ankle1* in murine tissues. (A) Representative end point PCR from tissue cDNA, *Actin* and *Gapdh* serve as loading control; (B) Quantitative real time PCR, samples are normalized to *Ankle1* expression in primary mouse embryonic fibroblasts (MEFs), 3 ≤ n ≤ 5, error bars: standard deviation.

Human ANKLE1 is most abundantly expressed in bone marrow and fetal hematopoietic tissues [2]. To test if *Ankle1*’s tissue specific expression is conserved in mouse, we isolated mRNA from tissues of 8 weeks old mice and performed semi-quantitative end point PCR (Fig 1A). Murine *Ankle1* expression is tissue specific, however less restricted compared to human tissues. Highest expression was detected in hematopoietic tissues including bone marrow, spleen and thymus, but colon, liver, ovary and testis also showed significant mRNA expression (Fig 1A and 1B). As shown previously [2], various antibodies that detect recombinant ANKLE1 expressed in mammalian cells failed to detect endogenous ANKLE1 protein in those cells and tissues that showed abundant *Ankle1* mRNA expression even after enrichment by immunoprecipitation, suggesting that ANKLE1 protein levels are extremely low and/or strictly regulated.

**Fig 2:**
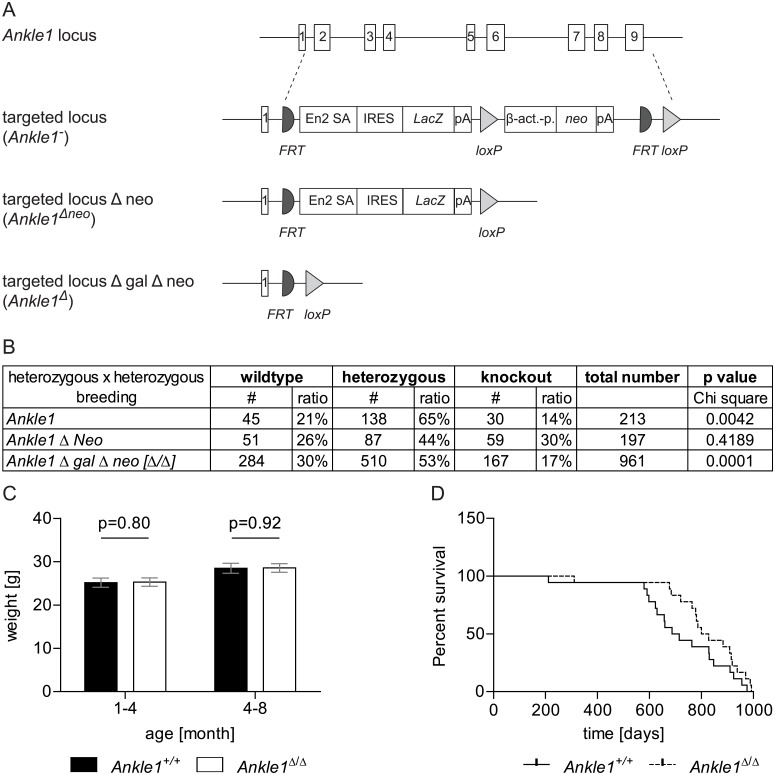
*Ankle1* knock-out mouse model. (A) Schematic drawing of the *Ankle1* wild-type locus with exons 1 to 9, the knock-out allele carrying the complete deletion cassette (*Ankle1*^*-*^) consisting of FRT recombination sites (semi circle), splice acceptors site (En2 SA), Internal Ribosome Entry Site (IRES), LacZ gene (*LacZ*), polyadenylation site (pA), loxP recombination site (triangle), β-actin promoter (β -act.-p.) and aminoglycoside phosphotransferase gene (neo) and the knock-out allele after CRE-recombination (*Ankle1*^Δneo^) carrying the reporter gene cassette only and the knock-out allele after FLP-mediated recombination (*Ankle1*^Δ^); (B) Birth statistics of different *Ankle1*-deficient mouse strains (for organization of the genomic locus see Fig 2A) from heterozygous pairings, for determination of statistical significant differences between the observed and the expected birth ratios (25% wildtype, 50% heterozygous, 25% knock-out) a Chi square test was performed, p≤0.05 statistical significant, p≤0.01 very statistical significant; (C) Weight of *Ankle1*^*+/+*^ and *Ankle1*^Δ/Δ^ animals 1–4 months and 4–8 months old, paired students t-test, statistical significance p≤0.05, 17 ≤ n ≤ 19; (D) Survival curve for *Ankle1*^*+/+*^ and *Ankle1*^Δ/Δ^ mice, log rank (Mantel-Cox) test p = 0.11, n = 18.

In order to get insight into potential *in vivo* functions of ANKLE1 in mice we generated an *Ankle1*-deficient mouse model. We obtained C57BL/6N embryonic stem (ES) cells from the EUCOMM project in which the *Ankle1* gene was constitutively inactivated by targeting the locus with a deletion cassette consisting of a *LacZ* reporter gene under the transcriptional control of the endogenous *Ankle1* promoter and a neomycin resistance gene under the control of a β-actin promoter (Fig 2A and S1A Fig). We confirmed the correct targeting of the construct to the *Ankle1* locus using long-range PCR (S1B Fig) in ES cells and in *Ankle1*^*+/-*^ mice and verified the single integration of the targeting cassette by Southern Blots against the neomycin resistance gene (S1C Fig). Subsequently, we generated an *Ankle1*-deficient mouse strain lacking the resistance cassette (*Ankle1*^Δneo^) by crossing the original *Ankle1*^*+/-*^ mice with C57BL/6J CRE deleter mice, and a strain lacking the complete deletion cassette (*Ankle1*^Δ^) by crossing with C57BL/6J FLP-deleter mice (Fig 2A and S1A Fig). The depletion of the cassettes by the recombinases was checked and monitored for several mouse generations by PCR (S1A and S1D Fig).

*Ankle1*-deficient mice from all three strains are viable and show no obvious defects, gain weight (Fig 2C) and have a life span (Fig 2D) similar to wild-type controls. However, in heterozygous pairings of *Ankle1*^*-*^ and *Ankle1*^Δ^ strains we observe a discrepancy between the observed and expected Mendelian birth ratio with reduced numbers of homozygotic *Ankle1*- deficient pubs, while the birth ratio of the strain carrying the *LacZ* reporter gene only (*Ankle1*^Δneo^) appeared normal (Fig 2B). In addition E 13.5 d *Ankle1*^Δ/Δ^ embryos of heterozygous pairings were undistinguishable from their littermates, and pairings of *Ankle1*^Δ/Δ^ mice that themselves were born in reduced numbers in the heterozygous pairings, yielded normal litter sizes of up to 12 pubs. Therefore we speculate that the threshold for embryonic lethality may also depend on small changes in the gene locus and /or genotype-independent factors.

Our expression analysis (Fig 1) showed that *Ankle1* is highest expressed in bone marrow, spleen and thymus, tissues that are essential for processes in hematopoiesis, such as hematopoietic stem cell differentiation and erythro- and lymphopoiesis. These processes require an efficient DNA repair machinery that includes several endonucleases [15–21]. We therefore tested, whether the endonuclease ANKLE1 is required in hematopoiesis by analyzing the composition of the hematopoietic progenitor repertoire, erythropoiesis and B and T cell development in the *Ankle1*-deficient mice using flow cytometry.

**Fig 3:**
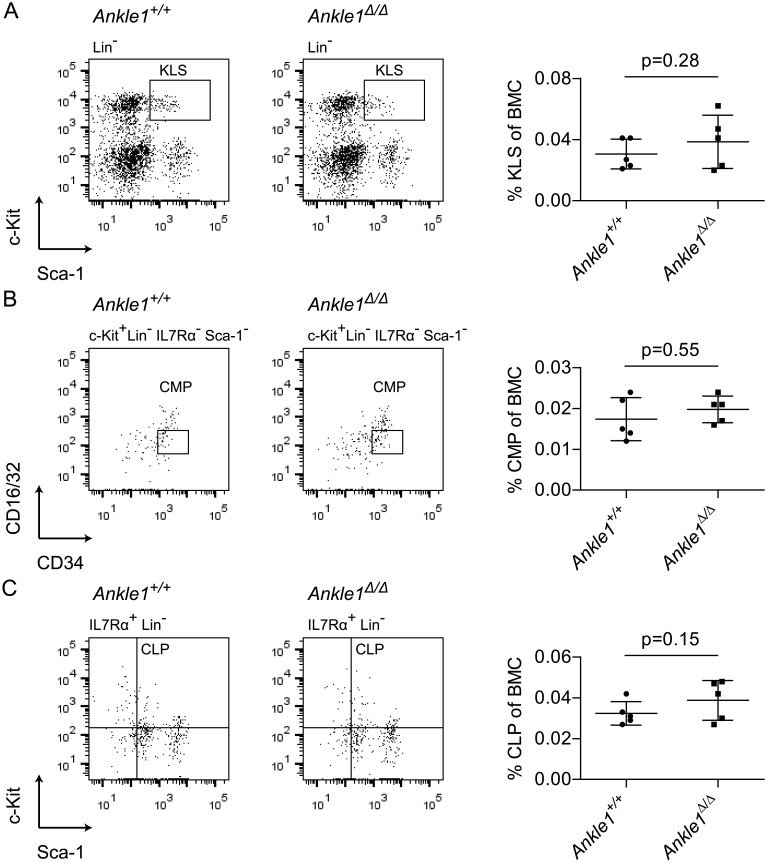
Flow cytometric analysis of early hematopoietic development in 9–10 months old *Ankle1*^Δ/Δ^ mice. (A) Representative dot plot of lineage negative bone marrow cells (BMCs) of an *Ankle1*^*+/+*^ and an *Ankle1*^Δ/Δ^ mouse and relative quantification of the KLS cells (c-Kit+ and Sca-1+, boxed) depicted in a scatter plot; (B) Representative dot plot of c-Kit+ Lin- IL7Rα- Sca-1- BMCs of an *Ankle1*^*+/+*^ and an *Ankle1*^Δ/Δ^ mouse and relative quantification of the boxed, CD16/32^low^ CD34^+^ common myeloid progenitors (CMPs) in a scatter plot; (C) Representative dot plot of IL7Rα^+^ Lin^-^ BMC of an *Ankle1*^*+/+*^ and an *Ankle1*^Δ/Δ^ mouse and relative quantification of the c-Kit^+^ Sca-1^+^, common lymphoid progenitors (CLPs) depicted in a scatter plot; scatter plots indicate the mean (n = 5), error bars: standard deviation, paired students t test was used for determination of statistical significant differences (p≤0.05).

First we tested, if the hematopoietic stem and progenitor cell repertoire is affected by loss of *Ankle1* in 9–10 months old mice (Fig 3). Bone marrow cells were stained with antibodies against lineage markers (Lin), c-Kit and Sca-1 to quantify KLS cells, representing a mixture of hematopoietic stem cells and progenitor cells [22]. In addition, we quantified common myeloid progenitors (CMP) (Fig 3B) and common lymphoid progenitors (CLP) (Fig 3C), which are often reduced in DNA damage repair-deficient mice [21]. All compartments showed a normal cell number as compared to the wild-type controls indicating that *Ankle1*-deficiency has little or no effect on hematopoietic stem and progenitor cell differentiation.

**Fig 4:**
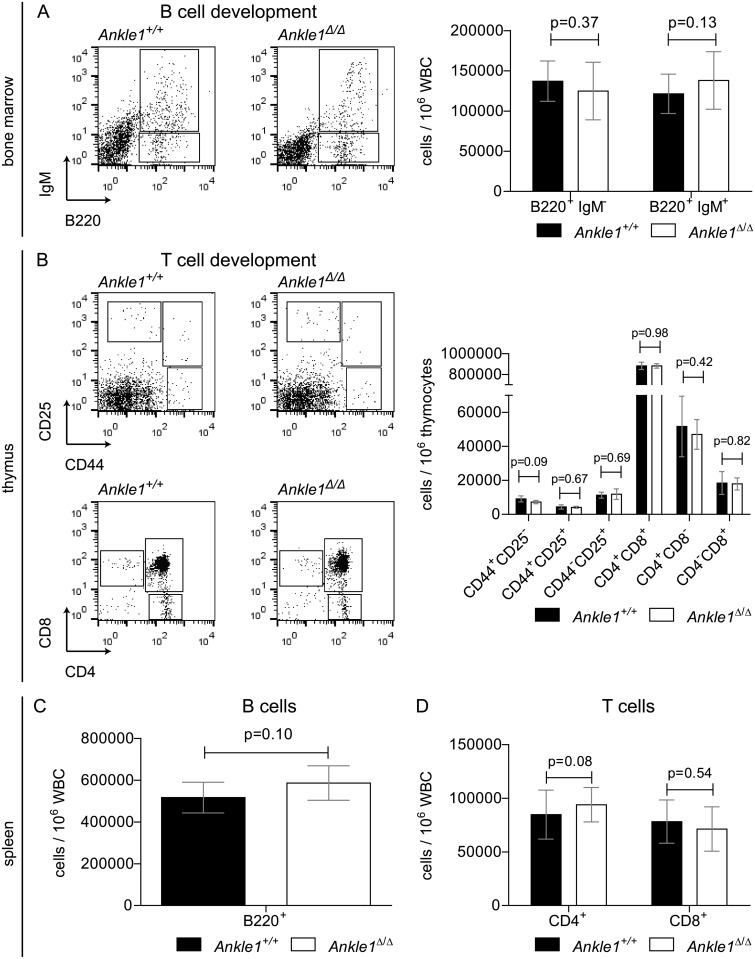
Flow cytometric analysis of lymphoid differentiation in 8 weeks old *Ankle1*^Δ/Δ^ mice. (A) Representative dot plots of B cell progenitors (B220^+^ IgM^-^) and B cells (B220^+^ IgM^+^) in white blood cells (WBC) from bone marrow of an *Ankle1*^*+/+*^ and an *Ankle1*^Δ/Δ^ mouse and relative quantification of the B220^+^ IgM^-^ and B220^+^ IgM^+^ cells in a bar graph; (B) Representative dot plots of consecutive developmental stages of T cell progenitors in thymus of an *Ankle1*^*+/+*^ and an *Ankle1*^Δ/Δ^ mouse and its relative quantification; Relative quantification of (C) B220^+^ and (D) CD4^+^ and CD8^+^ WBCs in spleen; error bars: standard deviation, paired students t test was used for determination of statistical significant differences (p≤0.05), n = 5.

B- and T cells both originate from the CLPs and are generated in bone marrow and thymus, respectively. During their differentiation both lineages undergo genomic rearrangements in their lymphoid receptor loci, requiring endonucleases for DNA cleavage and subsequent repair of the lesions [15, 20, 23]. Unsuccessful rearrangements lead to the depletion of the subsequent developmental stages. Furthermore, precursor B and T cells undergo a massive clonal expansion during their differentiation process, also requiring an efficient DNA repair machinery [16]. To test, whether these processes are affected in *Ankle1*-deficient mice, we quantified the number of B cell progenitors before and during the rearrangement process (B220^+^ IgM^-^) and immature B cells (B220^+^ IgM^+^) after rearrangement in the bone marrow (Fig 4A), as well as mature B cells in spleen (Fig 4C) in 8 weeks old mice. Precursor T cells were isolated from thymus and stained with CD25 and CD44 or CD4 and CD8 antibodies to determine the number of cells before, during and after the first and second T cell receptor chain rearrangement (Fig 4B). Also the frequency of mature CD4^+^ and CD8^+^ T cells in spleen was determined (Fig 4D). In all cases, the number of B and T cells at the analyzed developmental stages in *Ankle1*-deficient mice were comparable to those of their wild-type littermates, suggesting that B- and T cell development in a pathogen-free environment are not affected.

**Fig 5:**
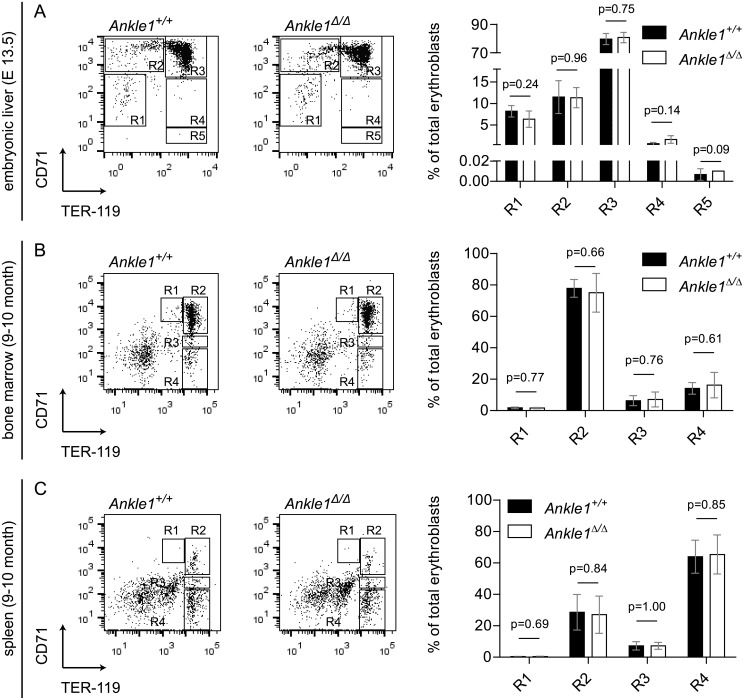
Flow cytometric analysis of erythroid differentiation in *Ankle1*^Δ/Δ^ mice. Cells were labeled with antibodies against Ter119 and CD71 (A) Representative dot plots of fetal liver cells from *Ankle1*^*+/+*^ and *Ankle1*^Δ/Δ^ embryos (E 13.5), regions 1–5 (R1–R5) represent five erythroid maturation stages, bar graphs show the percentage of labeled cells in each differentiation stage, n _litter_ = 3; Representative dot plots of (B) Bone marrow and (C) Splenic cells from an 9–10 months old *Ankle1*^*+/+*^ and an *Ankle1*^Δ/Δ^ mouse, regions 1–4 (R1–R4) represent four erythroid maturation stages, bar graphs show the percentage of labeled cells in each differentiation stage, n = 5; error bars: standard deviation, students t test was used for determination of statistical significant differences (p≤0.05).

Next we turned to erythroid lineage development. Erythropoiesis is located mainly in bone marrow but also in the spleen of adult mice and is strictly dependent on genomic integrity [17]. Fetal erythropoiesis starting from day 12.5 of embryonic development onward in liver shows a similar sequence of differentiation steps as in adult erythropoiesis. Stages of erythroid differentiation can be identified by the expression of the surface markers CD71 and TER119. We analyzed erythroblast differentiation in bone marrow and spleen in 9–10 months old mice by flow cytometry (Fig 5B and 5C). R1 to R4 populations represent four consecutive stages in erythroblast differentiation. Relative quantification of each stage did not reveal any signs of a differentiation block in erythropoiesis in *Ankle1*^Δ/Δ^ mice, indicating that ANKLE1 is not required for steady-state erythropoiesis in bone marrow and spleen. Embryonic erythropoiesis differs from steady-state erythropoiesis in adults due to the rapid growth of embryos requiring excessive generation of new erythrocytes to supply the newly formed tissues with sufficient oxygen, and resembles an *in vivo* genotoxic stress condition [24]. Because mild phenotypes in erythropoiesis often manifest in stress responses and we have observed a decrease in *Ankle1*-deficient pubs in litters of heterozygous parents, we hypothesized that ANKLE1 may be important for embryonic stress erythropoiesis. Hence we analyzed erythroblast differentiation in fetal liver on embryonic day 13.5. At this developmental stage only a few cells are fully differentiated and most erythroblasts are found in differentiation stage R3, in which CD71 is still highly expressed and TER119 fully upregulated (Fig 5A). Despite the high proliferation rates in embryonic erythropoiesis, we detected normal differentiation patterns in *Ankle1*-deficient embryos showing that ANKLE1 is not required in this process resembling geneotoxic stress conditions, and therefore unlikely to be the cause for the partial embryonic lethality of *Ankle1* knock-out mice in heterozygous pairings.

Altogether, we did not observe any defects in the hematopoietic system of *Ankle1*-deficient mice under normal and genotoxic stress conditions, although we cannot fully exclude that it may have a role in erythropoiesis under specific circumstances. Thus we concluded that ANKLE1’s role in normal erythropoiesis is redundant or that ANKLE1 has a hematopoiesis-independent function.

**Fig 6:**
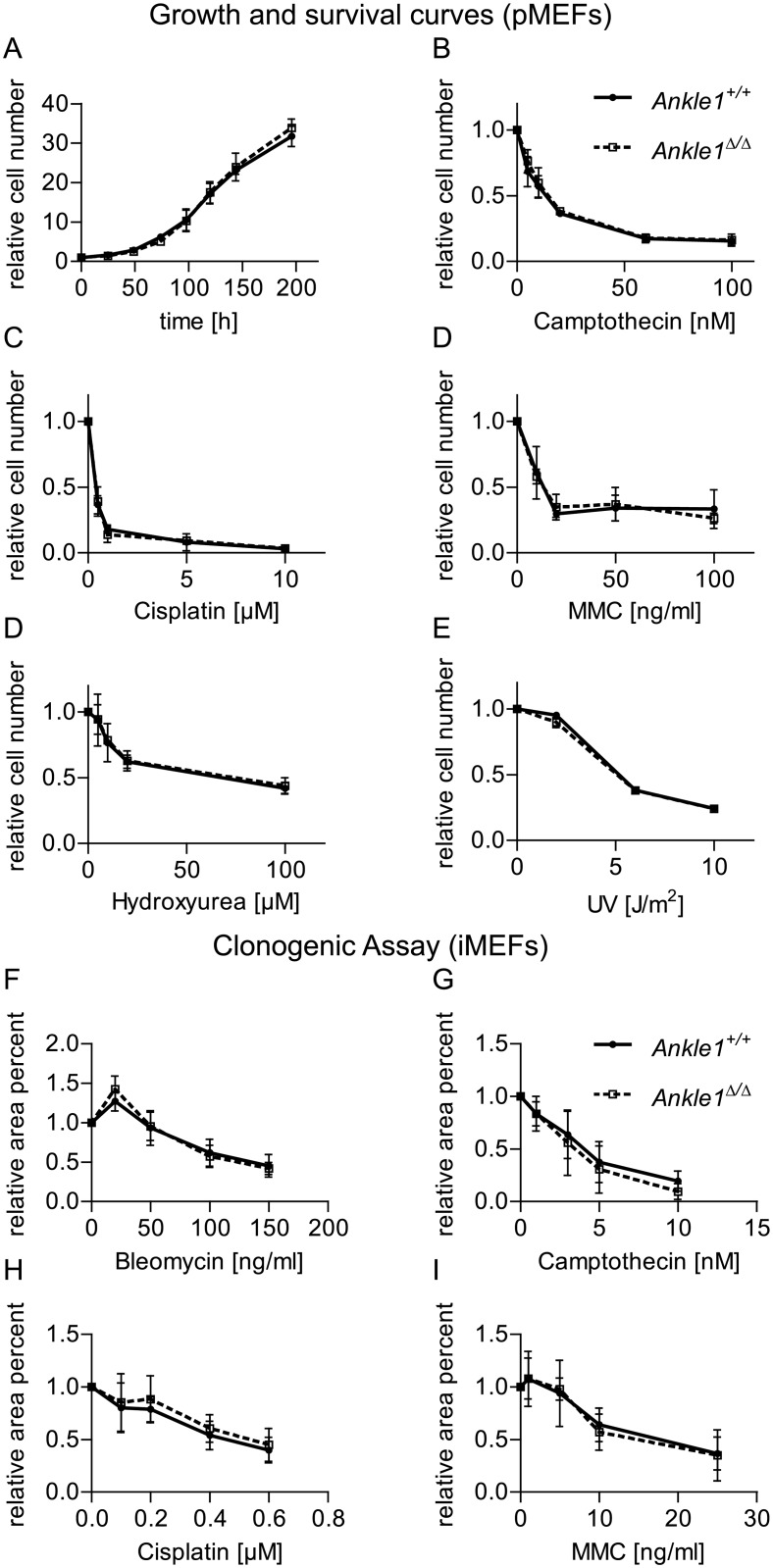
Growth and DNA damage sensitivity of *Ankle1*-deficient mouse embryonic fibroblasts (MEFs). (A) Growth curve of primary MEFs (pMEFs), n = 3, error bars: standard deviation; (B-D) Survival of pMEFs after chronic exposure to indicated DNA damaging agents for 72 h, n = 3, error bars: standard deviation; (E) Survival of pMEFs 72 h after single exposure to increasing doses of UV-light, n = 3, error bars: standard deviation; (F-I) Clonogenic survival assay of immortalized MEFs (iMEFs) after chronic exposure to indicated DNA damaging agents for 10–14 days, n = 2–3, error bars: standard error of the mean.

Small nucleotide polymorphisms within the human *Ankle1* gene have been associated with increased risk for certain forms of breast and ovarian cancer [25–27]. Given the fact, that ANKLE1 is a GIY-YIG endonuclease [1, 2, 8] and the only other so far identified protein with a GIY-YIG domain in mammals is the resolvase SLX1, which is involved in the resolution of Holliday junctions after HR, we hypothesized that ANKLE1 may have a similar resolvase function in DNA repair. First, we tested whether *Ankle1*-deficient cells are more sensitive towards genotoxic stress than wild-type controls. We isolated primary mouse embryonic fibroblasts (MEFs) (E13.5) and performed DNA damage response analyses after their first passage in culture. Without any treatment the growth rates of wild-type and *Ankle1*^Δ/Δ^ MEFs were undistinguishable within 4–5 cell divisions (Fig 6A). Exposure of the cells to Camptothecin, Cisplatin, Mitomycin and Hydroxyurea for 72 h as well as a single exposure to UV light after which the cells were cultured for 72 h leads to similar survival rates in wild-type and *Ankle1*-deficient cells (Fig 6B–6E). In order to monitor the response of the cells to long-term DNA damage we immortalized wild-type and *Ankle1* knock-out MEFs and performed clonogenic assays in the presence of Bleomycin, Camptothecin, Cisplatin and Mitomycin (Fig 6F–6I) over the course of 10 to 14 days, continuosly challenging the cells' DNA damage repair machinery. However, *Ankle1*-deficient MEFs did not exhibit an enhanced sensitivity towards any of the DNA damaging agents applied.

**Fig 7:**
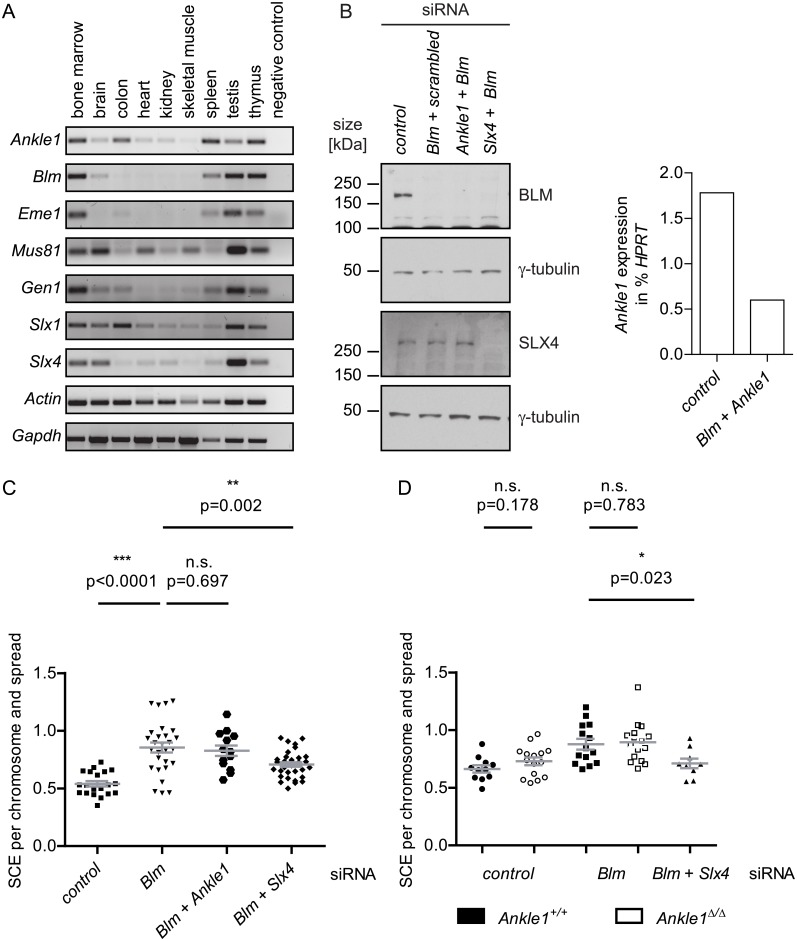
Involvement of ANKLE1 in Holliday junction resolution. (A) mRNA expression profile of *Ankle1* and known proteins involved in Holliday junction resolution after double strand break repair in somatic cells, representative end point PCR, *Actin* and *Gapdh* serve as loading control; (B) Western blot and qPCR analysis of iMEFs 48 h after transfection with indicated siRNAs; Scatter plot of sister chromatid exchange (SCE) frequencies in (C) Wild-type iMEFs and (D) Wild-type vs. *Ankle1*^Δ/Δ^ iMEFs following siRNA treatment, each dot represents one metaphase spread, horizontal bars represent the mean, error bars: standard error of the mean, p values were determined using the students t test, n.s. not significant, * significant (p≤0.05), ** very significant (p≤0.01), *** extremely significant (p≤0.001).

In parallel we challenged the hypothesis that as a resolvase, ANKLE1, like SLX1 may be involved in the resolution of Holliday junctions and in sister chromatid exchange (SCE). We first compared expression patterns of the components involved in Holliday junction resolution, including Bloom Helicase (*Blm*), *Eme1*, *Mus8*1, *Gen1*, *Slx1* and *Slx4* with that of *Ankle1* (Fig 7A). We found some commonalities in tissue-specific mRNA expression, such as the high expression of all tested genes in bone marrow and testis. However, the high *Ankle1* expression in spleen is unique within the tested genes.

To test whether ANKLE1 is involved in Holliday junction resolution, we performed SCE assays in immortalized MEFs. In somatic wild-type cells, double Holliday junctions are mainly dissolved by Bloom Helicase (BLM) without strand cleavage thereby avoiding SCE. In the absence of BLM, resolvases like SLX1, MUS81 and GEN1 introduce strand breaks, which lead to crossover or non-crossover events during the resolution of entangled sister chromatids. We depleted *Blm* levels by siRNA in MEFs to trigger the resolvase-dependent DNA repair pathway and tested under these conditions whether lack of *Ankle1* leads to a change in SCE rates. As a positive control we depleted SLX4, a well-characterized scaffold protein essential for SLX1 activity [13]. The knock-down of *Blm* and *Slx4* led to a strong reduction of respective protein levels, *Ankle1* mRNA was reduced by around 65% (Fig 7B). As expected, *Blm* depletion led to a significant increase in SCE rates compared to the control, while double knock-down of *Blm* and *Slx4* lowered this rate (Fig 7C). In contrast, *Ankle1* knock-down in a *Blm*-depleted background did not affect SCE rates, indicating that ANKLE1 has no major role in Holliday junction resolution in MEFs. Furthermore, knock-down of *Blm* in immortalized *Ankle1*^Δ/Δ^ MEFs led to a similar increase in the SCE frequency as compared to wild-type and *Ankle1*-knockdown MEFs (Fig 7D), ruling out that inefficient *Ankle1* mRNA knockdown was responsible for the unaffected SCE rates.

## Discussion

The aim of this study was to elucidate the in vivo function of the evolutionarily conserved GIY-YIG-type endonuclease ANKLE1 in mice. In a newly generated *Ankle1*-deficient mouse model we performed a comprehensive analysis of hematopoiesis, as *Ankle1* expression is tissue restricted and most abundant in hematopoietic tissues, such as bone marrow, spleen and thymus. Two previous reports suggested that ANKLE1 may be involved in DNA damage repair [1, 2]. hANKLE1 was demonstrated to have endonuclease activity *in vitro* and *in vivo* that nicks and cleaves double stranded DNA [2], and a *C*. *elegans* mutant strain bearing a catalytic inactive mutant of the ANKLE1 orthologue in worm, *lem-3*, was hypersensitive towards UV- and ionizing irradiation and the DNA crosslinking agent cisplatin [1]. These findings let us hypothesize that ANKLE1 may ensure DNA integrity during hematopoiesis in mice.

However, *Ankle1*-deficient mice neither showed abnormal numbers of hematopoietic progenitor cells, of common lymphoid and myeloid progenitors, nor did they reveal any impairment in lymphoid development and in adult erythropoiesis. In addition, embryonic erythropoiesis on E 13.5 d, considered to resemble stress erythropoiesis with increased genotoxic stress appeared normal. It is therefore possible that *Ankle1*-deficiency may be compensated by other DNA repair enzymes, particularly since many DNA damage repair pathways are redundant to ensure genome stability [28, 29]. Therefore, we cannot rule out that combined depletion of ANKLE1 and one or more other DNA repair components may uncover ANKLE1-specific defects. One candidate for enzymes functioning redundantly with ANKLE1 in DNA repair is the GIY-YIG containing resolvase SLX1 [8] involved in Holliday junction resolution during HR-mediated DNA repair [9]. SLX1 has partially redundant functions with two other known resolvases, MUS81 and GEN1 [11]. Interestingly, mice lacking SLX1 [13] or MUS81 [14] are viable, fertile and develop without major defects.

As *C*. *elegans* mutants lacking the three known resolvases SLX1, MUS81 and GEN1 retain some residual resolvase activity [12] potentially mediated by *lem-3*, the *C*. *elegans* orthologue of ANKLE1, we speculated that murine ANKLE1 may be a Holliday junction-resolving enzyme. Therefore, we tested if ANKLE1 is involved in the resolution of Holliday junctions in MEFs. However, unlike *Slx1*-deficient MEFs that are more sensitive to the DNA crosslinker mitomycin C compared to control cells [13], *Ankle1*-deficient MEFs did not reveal an increased sensitivity towards a number of different genotoxic agents. Furthermore, *Ankle1*-deficiency did not affect SCE in cells depleted for BLM, as reported for *Slx1*-deficient cells [13]. One possibility for the lack of a DNA damage repair phenotype in *Ankle1*-deficient MEFs may be the low expression of *Ankle1* in MEFs compared to the 5–10 times higher expression in hematopoietic cells. However, also stress erythropoiesis in *Ankle1*-deficient embryos was not affected, making it unlikely that application of genotoxic stress to *Ankle1*-deficient mice postnatally would have any considerable phenotype.

A potential role of ANKLE1 in DNA damage repair and hematopoesis is consistent with genome wide association studies showing that small nucleotide polymorphisms within the human *Ankle1* gene are associated with an increased risk for breast and ovarian cancer in BRCA1 mutation carriers, [25–27] and for multiple autoimmune syndromes [30]. However, *Ankle1*-deficient mice do not show increased predisposition to any malignancy and have a normal life span. One can only speculate about the reasons for these inconsistencies between mouse and man. The cancer-linked polymorphism in human ANKLE1 is located in its Ankyrin repeats, which are common protein-protein interaction motifs [31]. One may thus speculate that impaired protein interaction networks of ANKLE1 variants may lead to more severe deregulation of DNA repair pathways than the full depletion of *Ankle1* in mice. The short lifespan of mice compared to man may also contribute to the fact that *Ankle1*-deficient mice are not prone to cancer.

The only subtle phenotype we observed in two out of the three *Ankle1*-deficient mouse strains was a non-Mendelian birth ratio in heterozygous matings. However, homozygous mice are fertile and have normal sized litters. Therefore, we assume that this mild phenotype might either depend on small changes of the locus or on a weak, not yet identified meiotic defect.

Overall, the *Ankle1*-deficient mouse model gives little insight into the physiological and mechanistic function of the endonuclease ANKLE1. Nevertheless, research on ANKLE1 needs further attention due to the link to human cancer development. The most promising approach for these analyses will be to eliminate different, potentially compensatory components involved in DNA damage repair pathways in *Ankle1*^*-/-*^ mice, such as members of the BRCA1 complex and other Holliday junction-resolving enzymes.

## Materials and Methods

### mRNA expression analysis in murine tissue

RNA from murine tissues of 6–8 weeks old wild-type mice were isolated with TRIzol (Invitrogen) according to manufacturers protocol. Tissues were homogenized in a Precellys homogenizer (Peqlab). cDNA synthesis was performed according to manufactures’ protocols either with First Strand cDNA Synthesis Kit for RT-PCR (Roche) or M-MuLV Reverse Transcriptase (Fermentas) using 1 μg to 2 μg of total RNA and oligo-dT-primers.

End-point PCR for mRNA expression analysis of various genes was performed using GoTaq Green Master Mix (Promega) and the following primers:

Actin-f (5’ ATCTGGCACCACACCTTCTAC 3’),

Actin-r (5’ CAGCCAGGTCCAGACGCAGG 3’),

Ankle1-f1 (5’ GGCGGGAAGGCATTACAAAG 3’),

Ankle1-r1 (5’ TCGAGCCTGAATGTCCTGAG 3’)

Ankle1-f2 (5’ TGCCCTAGGTCTCCAGACAC 3’),

Ankle1-r2 (5’ GTCACATCCTCAGCATTCAG 3’),

Blm-f (5’ CTTTGAATTGTGGGACCGAATTG 3’),

Blm-r (5’ GTGGTGGGTAAACATTCCTCAG 3’),

Eme1-f (5’ GGAGATGGCCAGTGCTATTG 3’),

Eme1-r (5’ CCACGTCGCACTTGTATGTC 3’),

GAPDH-f (5’ CATCACCATCTTCCAGGAGCGA 3’),

GAPDH-r (5’ CCTGCTTCACCACCTTCTTGAT 3’),

Gen1-f (5’ TCTGCCCTTGTGAATGGCATC 3’),

Gen1-r (5’ TGGAATGGAAATCCTTCGCAAC 3’),

Mus81-f (5’ GTGGACATTGGCGAAACCAGA 3’),

Mus81-r (5’ CTCCAACGTGTAGCTTGCGT 3’),

Slx1-f (5’ CCGCCCTTCGGTTTGAATGG 3’),

Slx1-r (5’ TCGCTGTCAGCCTCGCTTAC 3’),

Slx4-f (5’ caggcatctaccactgagac 3’),

Slx4-r (5’ actcaaacgcagctccaaac 3’).

After amplification equal sample volumes were loaded on agarose gels and resolved via gel electrophoresis.

Quantitative real-time PCR for expression analysis in murine tissues was performed using 2x KAPA SYBR FAST QPCR MasterMix Universal (Peqlab), an Eppendorf Realplex 4 Mastercycler and primers Ankle1-f2 (5’ TGCCCTAGGTCTCCAGACAC 3’) and Ankle1-r2 (5’ GTCACATCCTCAGCATTCAG 3’) for *Ankle1* expression, as well as primers Hprt forward (5’ TGATTAGCGATGATGAACCAGG 3’) and Hprt reverser (5’ CTTTCATGACATCTCGAGCAAG 3’) for *Hprt* expression. Three technical replicates were averaged for each tissue. Relative *Ankle1* expression was evaluated via the ΔCt value method using *Hprt* as a reference gene and *Ankle1* expression in MEFs was set to 1. Tissues of two to four mice were analyzed. Data are represented as mean ± SD.

### Mouse strains and husbrandry

In order to generate *Ankle1*-deficient mice, ES cell clones with targeted *Ankle1* alleles (tm1(KOMP)Wtsi) were purchased from the Knockout Mouse Project Repository (https://www.komp.org). ES cell expansion, blastocyst injection and blastocyst implantation was performed at the Stem Cell Centre of the Institute of Molecular Biotechnology in Vienna. Chimeric mice were crossed to wild-type C57BL/6J mice and heterozygous offspring was either crossed to wild-type mice, Cre-deleter mice (C57BL/6J) [32], Flp-deleter mice (C57BL/6J) [33] or other heterozygous offspring. After deletion of loxP or FRT flanked fragments, the Cre and Flp alleles were outcrossed by pairing to wild-type mice.

Mice were maintained in accordance with the procedures outlined in the Guide for the Care and Use of Laboratory Animals, the “Charter of Fundamental Rights of the European Union”, the opinion of the “European Group on Ethics in Science”, and the “Protocol on the Protection and Welfare of Animals”. The Animal Research is covered by the National Austrian legislation (Law of Animal Experiments 2012 (“TVG-Tierversuchsgesetz”; Federal Law regulating the “Experimentation on living animals” BGBI. I Nr.114/2012) and the overriding EU and international legislation and codes of conduct. Additionally, animal experiments involving genetic manipulations are governed by the “Gentechnikgesetz” (GTG, 12.07.1994).

Chimeric mice were generated at the Institute of Molecular Pathology and the Institute of Molecular Biotechnology of the Austrian Academy of Sciences in compliance with the then current Austrian law 2005, BGBl. I Nr. 162/2005 (TVG 1989) and within a general valid project license (GZ M58/003079/2009/8) that was approved by the Office of the Vienna provincial government on 10.07.2009. The license also permitted breeding, housing, and euthanasia of mice. The scope of this general license was the generation of chimeric mice by blastocyst transfer and included a total of 9000 mice. Mice were sacrificed by cervical dislocation. For this study 8 recipient mice were used within the general license and chimeras were transferred to the animal facility of the Max F. Perutz Laboratories (MFPL).

Starting with 2011, all further mouse breeding and housing of mice was done at MFPL according to the then effective Austrian Law (Tierversuchsgesetz 1989, BGBl. I Nr. 162/2005 TVG 1989). In contrast to the current Austrian law (Law of Animal Experiments 2012, BGBI. I Nr.114/2012) the older version effective during the time of generation of the transgenic animals imposes no separate and additional approval for animal experiments by the Federal Ministry for Science and Research (BMWF) besides the general permission of the Institute of Molecular Biotechnology shown above, nor ethical approval for the production and breeding of new transgenic mouse lines by the Animal Experiment Evaluation Committee of the Medical University (“Ethikkommission zur Beratung und Begutachtung von Studien am Tier der Medizinischen Universität Wien“. As the generated transgenic mice had no overt phenotype, did not show any signs of distress (see below) and only served as donors for tissues and cells in this study no specific permission from the Federal Ministry for Science and Research (BMWF) was required for this study. The operation of the MFPL mouse facility, where the transgenic mice were housed and bred was approved by the Austrian Federal Ministry of Science and Research (GZ) BMWFW-66.006/0012-WF/II/3b/2014. This general license permits breeding, housing, and euthanasia of mice by carbon dioxide. For this study a total of 1400 mice was bred and housed at the MFPL facility. Of these 1400 mice, 150 mice were used for breeding and generation of the different genotypes outlined in the manuscript, 38 animals for weight gain analyses, 36 mice for survival analysis, 11 mice for RNA isolation, 10 mice for isolation of hematopoietic progenitors and adult erythropoiesis analysis, 16 mice for the isolation of MEFs and 16 mice for the analyses of embryonic erythropoiesis. Most of these mice and additional 1200 mice were used for generating birth statistics of the different lines described in the manuscript. Mice were euthanized using carbon dioxide. With regard to animal welfare, animals were daily monitored for signs of distress as defined in the Report of the Federation of European Laboratory Animal Science Associations (FELASA) Working Group on Pain and Distress accepted by the FELASA Board of Management November 1992. These include crouched posture, inability to move and to reach food and water, but also self-inflicted injuries (e.g. by scratching), extreme disquiet or listlessness. None of the transgenic animals showed any of these symptoms and showed any overt phenotype compared to wild-type controls. Mice were euthanized by carbone dioxide at different ages as outlined in the manuscript to serve as cell donors for in vitro experiments, or they were kept in cages until natural death occurred.

Long range PCR: Mouse tail DNA used for long range PCRs and genotyping was extracted from mouse tail clips or where indicated from ES cells. Amplifications at the junctions between targeting cassette and endogenous locus were performed using Herculase II Fusion DNA Polymerase (Agilent Technology) and primers Ankle1-ko-targ-f1 (5’ GCCCTACTTCAGTGACGATG 3’) and Ankle1-ko-targ-r2 (5’ CCCTTCCTCCTACATAGTTG 3’) for the 5’ end. At the 3’ junction primers PNF (5’ ATCCGGGGGTACCGCGTCGAG 3’) and GR3 (5’ GCCTATTCAGATCAGGCGTGCTATATAG 3’) were used. The myogenin locus served as a control for DNA quality and loading (MyogenF 5’ TTACGTCCATCGTGGACAGC 3’, MyogR246 5’ TGGGCTGGGTGTTAGCCTTA 3’).

Southern Blot: Genomic DNA from murine liver was isolated using Omniprep for mouse tails (GBioscience). Southern Blot analysis was performed using standard protocols. DNA was digested using AflII and BseRI. The neomycin-resistance cassette probe template was PCR amplified using the primers Ankle1-ko-20714f (5’ AGCACAGAGCCTCGCCTTTG 3’) and Ankle1-ko-21330r (5’ CATGGGTCACGACGAGATCC 3’) and genomic ES cell DNA was cloned into the pCR2.1 plasmid (Invitrogen) and verified by sequencing. Before labeling the probes, the plasmid was digested with EcoRI and the bands were eluted from the gel. The Rosa 26 locus (Rosa26 5’ probe) served as a loading control [34]. The probes for hybridization were generated using Prime-It II Random Primer Labelling Kit (Agilent Technologies) and radioactively labeled [^32^P]dCTP (3000Ci/mmol) (Hartmann Analytic). The membrane was exposed to X-ray sensitive Kodak BioMax MR film (Sigma-Aldrich) for several days at −80°C.

Genotyping of mouse strains: Genomic DNA was prepared from tail tips and PCR analyses were performed using KAPA Mouse Genotyping Hot Start Kit (Peqlab) and the following primers: Ankle1-ko-20714 (5’ AGCACAGAGCCTCGCCTTTG 3’), Cresense (5’ CCAATTTACTGACCGTACACC 3’), Creantisense (5’ TAATCGCCATCTTCCAGCAGG 3’), Flpsense (5’ GTGGATCGATCCTACCCCTTGCG 3’), Flpantisense (5’ GGTCCAACTGCAGCCCAAGCTTCC 3’), gmAnkle1-17771f (5’ CAGCTGATCCTCTACTATCC 3’), gmAnkle1-17968r (5’ GCTCGGATACCTTTCATGTG 3’), KOMP-5’F (5’ TGGTCCTCTCAAAGCTCTAC 3’), KOMP-3’R2 (5’ GATGGCTCAGGATTCATGTC 3’), KOMP-en2-R (5’ CCACTGACCTTGGGCAAGAACAT 3’).

### Cell culture, MEF isolation and immortalization

Cells were cultured in DMEM supplemented with 10% to 20% FCS, 2 mM L-glutamine, non- essential amino acids and penicillin/streptomycin at 37°C in a humidified atmosphere containing 8.5% CO2.

For MEF isolation, 13.5 dpc embryos were derived from timed matings between heterozygous or homozygous parents. After decapitation, the heads were used for genotyping. The red organs were removed, the embryo was minced and resuspended in 1 ml trypsin/1mM EDTA and incubated at 37°C for 10 min before the addition of 10 ml growth medium. Cells were plated and allowed to attach over night before cells were washed with fresh medium to remove debris. When cells reached confluency they were frozen (1:3). Replating after thawing was considered passage 1. All experiments with primary MEFs were performed within passages 2 to 3. MEFs were immortalized via expression of SV40 large T antigen.

For analyses of cell growth, 3 x 10^4^ primary MEFs per well in a 6 Well plate of three independent cell lines for each genotype were seeded in quadruples for the first count (0 h) and in duplicates for the following time points. Cells were allowed to attach for 24 h and were then harvest and counted every day for 8 days in a row. First count corresponds to 0 h. Quadruples and duplicates were averaged and results were normalized to 0 h.

For testing cell survival, 1 x 10^5^ primary MEFs per well in a 6 well plate of three independent cell lines for each genotype were seeded in duplicates. 24 h after seeding fresh medium with the indicated concentration of genotoxin (Camptothecin, Cisplatin, Mitomycin C (MMC) and Hydroxyurea) was added. For UV-irradiation cells were washed with PBS and exposed to a UV-C source. After a 72 h incubation, cells were counted. Duplicates were averaged and cell numbers were normalized to the untreated sample.

For clonogenic assays, immortalized MEFs of three independent cell lines per genotype were seeded in triplicates on 6 well dishes and were allowed to attach. Fresh medium with the indicated concentration of genotoxin (Bleomycin, Camptothecin, Cisplatin, Mitomycin C (MMC)) was added. After 10 to 14 days cells were fixed and stained (0.05% crystal violet, 1% methanol, 1% paraformaldehyde in PBS). Plates were scanned and evaluated with the ImageJ-plugin “ColonyArea” [35]. Results were normalized to the untreated sample and triplicates were averaged. Experiments for each cell line were repeated 2 to 3 times and averaged. Data are represented as mean ± SEM.

### Flow cytometric analyses of hematopoietic cells

For isolation of embryonic erythroid cells, 13.5 dpc embryos were derived from timed matings between heterozygous or homozygous parents. After decapitation, the heads were used for genotyping. The liver was removed and resupended in medium and squashed through a 40 μm cell strainer, centrifuged and resuspended in PBS/2% BSA. Cells were incubated with Fc block (CD16/CD32, eBioscience) and rat serum (eBioscience) for 5 min and stained for 30 min. Samples were washed and measured. Results of the same genotype within one litter were averaged, 3 litters were analyzed and significance (p≤0.05) was determined by performing students t test (unpaired, two tailed). Data are represented as mean ± SD.

Isolation of hematopoietic cells from adult mice: Flow cytometric analyses of murine erythroid and early hematopoietic cells (KLS, CMP, CLP) were performed using 9 to 10 months old *Ankle1*^Δ/Δ^ mice and *Ankle1*^*+/+*^ littermates. For analyses of the lymphoid lineage, littermates were 8 weeks old. Bone marrow cells were isolated by flushing tibia and femur of both hind legs with PBS/2% BSA and filtering the cell suspension through a 40 μm cell strainer. Thymus and spleen were washed in FACS buffer and also filtered through a 40 μm mesh to obtain a single cell suspension of thymocytes and splenocytes. Cells were incubated with Fc block (CD16/CD32 clone 93, eBioscience) except for CMP samples and rat serum (eBioscience) for 5 min, stained with antibodies for 30 min. Samples were washed and measured. 5 littermates were analyzed and significance (p≤0.05) was determined by performing students t test (paired, two tailed). Data are represented as mean ± SD.

BD FACSCalibur was used for acquisition of lymphoid and erythroid samples, early hematopoietic cells were measured with BD FACS Aria. Data were evaluated using FlowJo (Tree Star Inc.).

The following antibodies were used for flow cytometric analyses: B220 FITC (clone RA3-6B2), CD4 FITC (clone GK1.5), CD8 PE (clone 53–6.7), CD16/CD32 PerCP-Cy 5.5 (clone 93), CD25 PE (clone PC61.5), CD34 FITC (clone RAM34), CD44 FITC (clone IM7), CD71 PE (clone R17217), c-Kit APC (clone 2B8), IgM PE (clone eB121-15F9), IL7Rα (CD127) FITC and PE (clone A7R34), Sca-1 PE (clone D7), TER-119 FITC (clone TER-119) and according isotype controls all from eBioscience as well as the PE anti-mouse lineage cocktail with isotype control from BioLegend.

### Sister chromatid exchange assay

For siRNA mediated knockdown of *Blm*, *Slx4*, *Ankle1* and control transfection 2.5 x 10^5^ MEFs/6-well were plated 8–12 h prior to the first siRNA transfection. 2 transfections 24 h apart were performed using 40 pmol ON-TARGETplus SMARTpool siRNA for each target and 5 μl DharmaFECT 1, all purchased from Dharmacon. Cell culture media was changed at the time of both transfections. The following siRNAs were used: ON-TARGETplus Non-targeting Pool (D-001810-10), SMARTpool: ON-TARGETplus Mouse Blm siRNA (L-061987-00), SMARTpool: ON-TARGETplus Mouse Slx4 siRNA (L-057081-01), SMARTpool: ON-TARGETplus Mouse *Ankle1* siRNA (D-001810-10-20). 48 h after the first transfection cells were harvested, 500 000 cells/10 cm were replated and 2x10^-4^ M BrdU was added to the medium. Remaining cells were lysed for protein and mRNA analysis.

The sister chromatid exchange assay was performed as described by Bayani et al. [36] with slight adaptions. After two days of BrdU incubation 0,1 μg/ml Colcemid was added to the cells for 45–60 min and thereafter mitotic cells were harvested, resuspended in 75 mM KCl and incubated for 15 min at 37°C, stepwise fixed using Methanol/acetic acid fixative (3:1) and stored at least over night at -20°C. Chromosomes were spotted on glass slides, slides were dried and rehydrated in PBS, stained for 5 min with Hoechst 33258, washed in PBS 2x SSC and exposed to UV-light for 15 min, slightly covered with 2xSSC and finally stained for 3 min in 4% Giemsa/Gurr buffer (pH 6.8), wash in distilled water and mounted in Eukitt (Sigma-Aldrich). Pictures of chromosome spreads were acquired using a Zeiss light microscope (magnification) and Axiovison Version.

For protein analysis harvested cells were counted, washed in PBS and resuspended in lysis buffer (8 M Urea, 25 mM Tris-HCl pH 7.4, 100 mM NaCl, 10 mM DTT, 1x complete protease inhibitor mix (Roche)) with a concentration of 20 000 cells/μl. 1 U/μl Benzonase was added and lysates were incubated for 15 min on ice. Lysates were mixed with Laemmli sample buffer, incubated for 20 min on 37°C and resolved via polyacrylamide gel electrophoresis. Proteins were blotted onto nitrocellulose membranes and probed with the following antibodies: BLM (A300-572A, Bethyl), SLX4 [13] (a gift from J. Rouse), γ-tubulin (GTU-88, Sigma-Aldrich). After incubation with horse-radish peroxidase conjungated secondary antibodies, the Amersham ECL Prime Western Blotting Detection Reagent and CL-XPosure films (Thermo Scientific) were used to detect signals.

For mRNA analysis the SV Total RNA Isolation System (Promega) was used to isolate total RNA from harvested cells according to manufacturer’s protocol and cDNA was synthesized using RevertAid Reverse Transcriptase (Thermo Scientific). Quantitative real-time PCR was performed using *Ankle1* and *Hprt* primer described above. Relative *Ankle1* expression was evaluated via the ΔCt value method using *Hprt* as a reference gene.

## Supporting Information

S1 Fig Disruption of the murine *Ankle1* locus. (PDF)
