# Supplementary material for: The GIY-YIG Type Endonuclease Ankyrin Repeat and LEM Domain-Containing Protein 1 (ANKLE1) Is Dispensable for Mouse Hematopoiesis
Source: PLoS One. 2016 Mar 24;11(3):e0152278. doi: 10.1371/journal.pone.0152278 (PMC4807109; doi:10.1371/journal.pone.0152278)
Supplement: S1 Fig — (PDF) [file pone.0152278.s001.pdf]

S1 Fig. Disruption of the murine *Ankle1* locus (A) Schematic drawing of the *Ankle1* wild-type locus with exons 1 to 9, the knock-out allele carrying the complete deletion cassette (*Ankle1*$^{-}$) consisting of 5' and 3' homology arms including flanking gene (blank box), FRT recombination sites (semi circle), splice acceptors site (En2 SA), Internal Ribosome Entry Site (IRES), LacZ gene (*LacZ*), polyadenylation site (pA), loxP recombination site (triangle), β-actin promoter (β -act.-p.) and aminoglycoside phosphotransferase gene (neo) and the knock-out allele after cre-recombination (*Ankle1*$^{\Delta neo}$) carrying the reporter gene cassette only and the knock-out allele after Flp-mediated recombination (*Ankle1*$^{\Delta}$), primer binding sites for the long range PCR (5' forward/reverse and 3' forward/reverse) (panel B) and genotyping PCR (1-6) (panel D) are indicated with arrows, binding site for the Southern blot neomycin probe (Neo probe) as well as the cleavage sites used for genome fragmentation (panel C) are marked; (B) Long range PCR with genomic DNA from ES cells and mouse tails to determine if targeted integration had occurred by homologous recombination, Primer 5' forward and 5' reverse, and Primer 3' forward and 3' reverse (for binding side see panel A) were used, Primer in the myogenin locus served as control; (C) Southern blot of extracted liver DNA of *Ankle1*$^{+/-}$ and *Ankle1*$^{+/+}$ mice digested with AflII and BseRI and probed against the neomycin resistance cassette for determination of potential double integration, probing against the Rosa locus served as loading control; (D) PCR analysis of genomic mouse tail DNA for analysis of successful cre- or flp-mediated recombination and for genotyping, primer binding sites are indicated in panel A

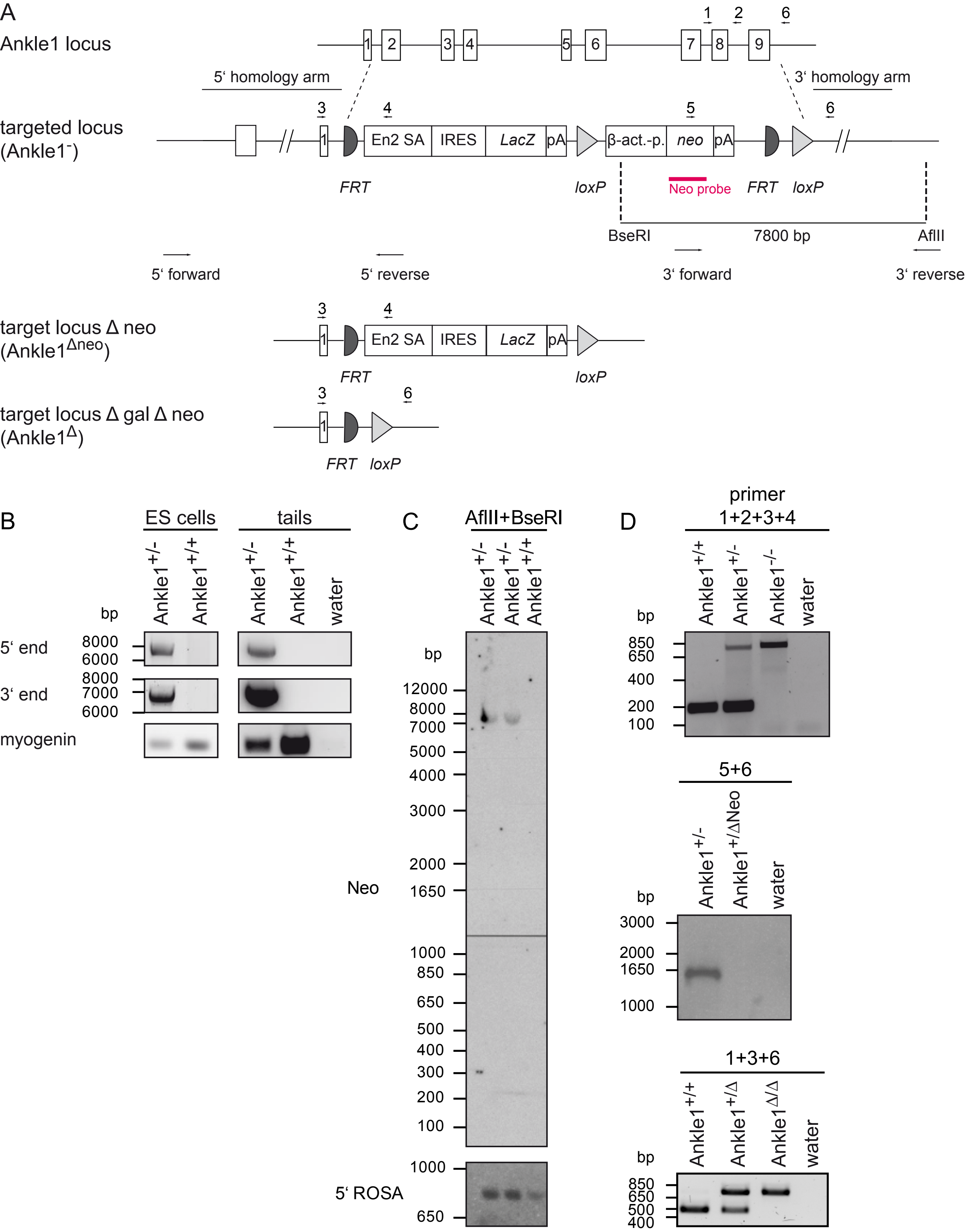

A
Ankle1 locus
1 2 6
1 2 3 4 5 6 7 8 9
5' homology arm
3' homology arm
3 4 5 6
targeted locus
(Ankle1-)
En2 SA
IRES
LacZ
pA
β-act.-p.
neo
pA
FRT
loxP
Neo probe
FRT
loxP
BseRI
7800 bp
AflII
5' forward
5' reverse
3' forward
3' reverse
target locus Δ neo
(Ankle1Δneo)
FRT
loxP
target locus Δ gal Δ neo
(Ankle1Δ)
FRT
loxP
B
ES cells
tails
Ankle1+/-
Ankle1+/+
Ankle1+/-
Ankle1+/+
water
bp
5' end
8000
6000
3' end
8000
7000
6000
myogenin
C
AflII+BseRI
Ankle1+/-
Ankle1+/-
Ankle1+/+
bp
12000
8000
7000
5000
4000
3000
2000
1650
Neo
1000
850
650
500
400
300
200
100
1000
5' ROSA
650
D
primer
1+2+3+4
Ankle1+/+
Ankle1+/-
Ankle1-/-
water
bp
850
650
400
200
100
5+6
Ankle1+/-
Ankle1+/ΔNeo
water
bp
3000
2000
1650
1000
1+3+6
Ankle1+/+
Ankle1+/Δ
Ankle1Δ/Δ
water
bp
850
650
500
400
